# Chromosome‐Level Genome Assembly and Whole‐Genome Resequencing Revealed Contrasting Population Genetic Differentiation of Black Bream (*Megalobrama skolkovii*) (Teleostei: Cyprinidae) Allopatric and Sympatric to Its Kin Species

**DOI:** 10.1002/ece3.70874

**Published:** 2025-01-21

**Authors:** Ruijin Ding, Dan Yu, Ke Yang, Xinghua Wu, Huanzhang Liu

**Affiliations:** ^1^ State Key Laboratory of Breeding Biotechnology and Sustainable Aquaculture, Institute of Hydrobiology Chinese Academy of Sciences Wuhan China; ^2^ College of Advanced Agricultural Sciences University of Chinese Academy of Sciences Beijing China; ^3^ Research Center for Yangtze River Ecological and Environmental Engineering China Three Gorges Corporation Beijing China

**Keywords:** ancestral polymorphism, black bream, founder effects, population bottlenecks, population differentiation, population genomics

## Abstract

The black bream ( *Megalobrama skolkovii* ) is an economically important species widely distributed in China, with its geographic populations potentially having undergone differentiations and local adaptations. In this study, we presented a chromosome‐level genome assembly of this species and investigated genetic differentiations of its populations that are allopatric (the northern one) and sympatric (the Poyang Lake) to its kin species, the blunt‐snout bream ( *M. amblycephala* ), using whole genome resequencing analysis. The results showed that the genome size of black bream was 1.13 Gb, very similar to its kin species but larger than its close relatives, the four Chinese major carps. By resequencing individuals from the northern and Poyang Lake populations, we found that the northern population showed lower genetic diversity, larger genetic differentiation, and two sharp historical declines in population size through demographic analysis, indicating the possible bottlenecks after the allopatric isolation. In contrast, the Poyang Lake population, with its higher genetic diversity, higher Tajima's *D* value, and lower levels of linkage disequilibrium, reflects the ancestral state of black bream. In addition, we also found that the northern population shared more alleles with its kin species, indicating it may retain more ancestral variations. This was further analyzed to be caused by incomplete lineage sorting and ancient introgression. Some key genes related to reproductive processes, body size development, and muscle metabolism were found under selection in the northern population, possibly responsible for its local adaptation. Our findings that the black bream allopatric population had a loss of genetic diversity but retained more ancestral variations can expand our knowledge on population genetic differentiation and give us hints for future genetic conservation.

## Graphical abstract

The chromosome‐level genome of the black bream ( *Megalobrama skolkovii* ) was assembled. Whole genome analysis at the population level revealed lower genetic diversity but more ancestral alleles in the northern population, while the Poyang Lake population exhibited higher genetic diversity but fewer ancestral alleles, despite its ancestral state.

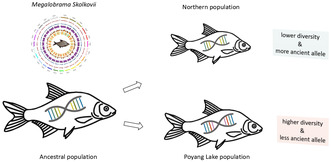

## 1 Introduction

Population genetic differentiation is often regarded as an early stage of speciation, which may lead to further divergence and eventually result in speciation, forming the source of biological diversity. Therefore, investigations on population genetic differentiation have drawn great attention in evolutionary biological studies. Many studies emphasized the importance of founder effects accompanied with allopatric or peripatric differentiations, where a small group of individuals becomes separated from the original population and creates a genetic bottleneck that speeds up the emergence of new combinations of alleles through the process of genetic drift (Mayr 1954; Hunter et al. 2010; Pastor et al. 2004; Roques and Negro 2005; Jangjoo et al. 2016; Weber, Stewart, and Lehman 2004). In these cases, due to bottleneck effects, founder populations may suffer a severe reduction in genetic diversity following colonization, resulting in lower nucleotide diversity and higher levels of linkage disequilibrium (Shultz et al. 2016; Templeton 2008; Carson and Templeton 2014; Barton and Charlesworth 1984). In contrast, ancestral (source) populations may retain high genetic diversity and exhibit low levels of linkage disequilibrium.

Normally, due to bottleneck effects or natural selection, most alleles in the genome were found to exhibit congruent patterns along with population differentiation. And the gene trees are usually congruent with species trees. However, ancestral polymorphism may lead to incomplete lineage sorting (ILS) in population differentiation, causing conflicting patterns between gene trees and species trees. Besides, introgression may occur after population divergence, also causing the conflicting patterns between gene trees and species trees (Tomasco et al. 2022; Ma, Ren, and Sun 2024; Edelman and Mallet 2021; Suvorov et al. 2022). Investigations on the results of founder effects, identifying the importance of ILS and introgression in population genetic differentiation, may deepen our understanding of the complexities of the forming of biological diversity and taking appropriate conservation measures.

In recent decades, with the propositions of different theories and the development of new methods and techniques, especially the increasing ubiquity and affordability of next‐generation sequencing technologies, our knowledge of population differentiation has greatly expanded (Vijay et al. 2016; Wang et al. 2016; Wolf and Ellegren 2017). For example, through the use of genomic data, founder effects and bottlenecks have been identified in many cases, leading to reductions in heterozygosity, high linkage disequilibrium, and elevated deleterious mutation loads in both animal and plant species (Kumar et al. 2023; Pilot et al. 2014; Shultz et al. 2016; Flanagan et al. 2021). Studies has revealed that ILS is pervasive in primate speciation and selection (Rivas‐Gonzalez et al. 2023; Hobolth et al. 2011). Introgressions in *Heliconius* butterflies have been found leading to the adaptive gene flow of wing pattern alleles across species boundaries, contributing to homoploid hybrid speciation (Brower 2013; Zhang et al. 2016; Edelman et al. 2019). These findings have greatly expanded our knowledge of population genetic differentiation and have also demonstrated the power of the new genomic methods as well.

**FIGURE 1:**
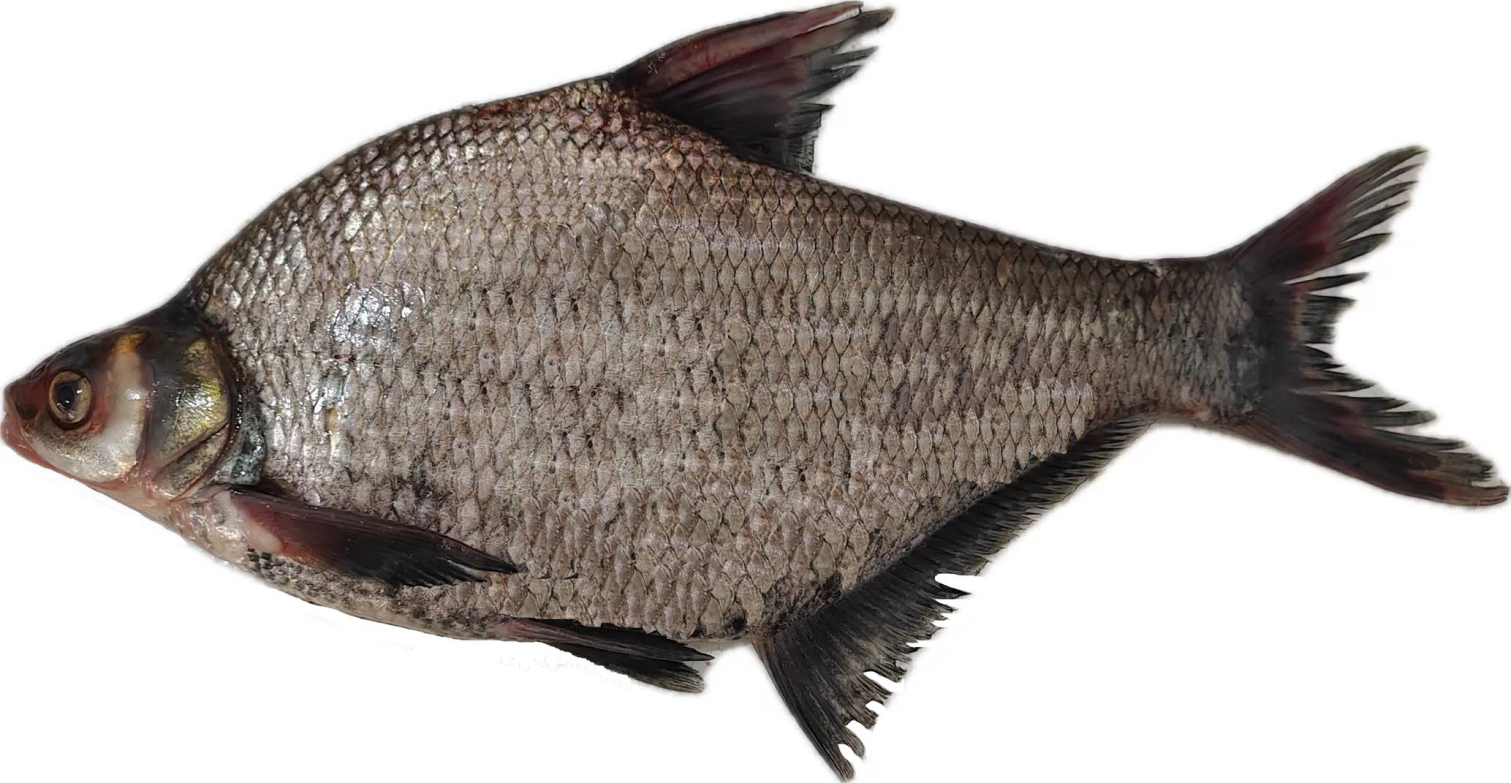
Photograph of black bream ( *Megalobrama skolkovii* ).

**FIGURE 2:**
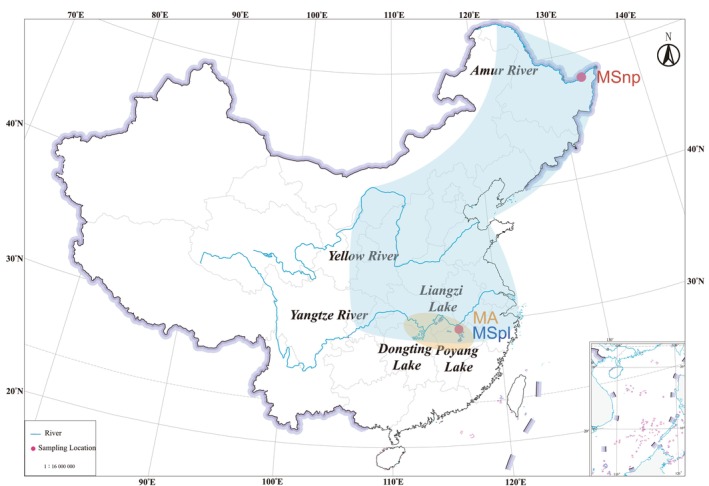
Distribution and sampling locations of black bream and *M. amblycephala* in China. Sampling sites are marked with red dots. MSnp and MSpl represent black bream populations from the Heilongjiang River and Poyang Lake, respectively; MA represents the *M. amblycephala* population from Poyang Lake. Major rivers and lakes are labeled to illustrate the geographical range.

The genus *Megalobrama*, belonging to the subfamily Cultrinae in Cyprinidae, holds a prominent position in China's economically vital fish species. At present, four species are identified in this genus, *M. amblycephala, M. skolkovii, M. pellegrini, and M. terminalis* (Yih 1955; Chen 1998a; Luo 1990), with the blunt‐snout bream ( *M. amblycephala* ) predominantly found in middle and lower Yangtze River lakes, while the black bream ( *M. skolkovii* ; Figure 1) is widely distributed in the Heilongjiang River, Yellow River, Yangtze River, Minjiang River, etc. (Figure 2; Chen 1998a; Mulian 1994; Luo 1990; Banarescu, Wu, and Banarescu 1967).

For the geological background and evolutionary history of East Asian freshwater fishes, it was suggested that, in the early Miocene (22.5 MYA), with the formation of the ancient Yangtze River and the appearance of monsoon climate, the early fish groups evolved. Then with the strengthening of monsoon climate (18.5 MYA), the lake–river complex system formed, with some fish groups adapted to lake habitats appearing (Cheng, Yu, et al. 2022; Feng et al. 2022; Chen 1998b). It is normally recognized that, even though the very long history of the lake–river complex system, for a specific lake, its history is usually not very long, maybe several thousand years. These lakes just kept disappearing in one place and appearing in another place in the geological history. And then, with the appearance of climate cooling and glaciation from late Pliocene, the cycling of glaciation and interglaciation drove the fishes dispersed from the Yangtze River (the centers of origin) to the northern part of China, forming the present distribution pattern (Ehlers, Gibbard, and Hughes 2018).

Due to the wide distributions, geographic populations of black bream have been found with distinct differentiations. It has been reported that the northern populations of black bream exhibit distinct morphological characteristics compared with the southern populations, including variations in the number of vertebrae, length of the intestine/body length, body length/eye diameter, and hypopharyngeal teeth type (Wang et al. 1993). The Heilongjiang River population was observed with reduced genetic diversity by mitochondrial DNA data, and significant genetic differentiations were revealed by the pairwise *F*st values among populations from the Heilongjiang River, the Qiantang River, and the Jinsha River (Hu, Ma, et al. 2020). Even though there are enormous works on the morphological and genetic studies on the *Megalobrama* species, population differentiations of the black bream have not been investigated extensively.

In this study, we generated a high‐quality, chromosome‐level genome assembly of black bream by integrating data from PacBio, Hi‐C, and MGISEQ technologies. Then, by conducting whole‐genome resequencing on 39 samples from the Heilongjiang River (northern China) and the Poyang Lake, we analyzed genomic variations in these two populations to compare the genomic divergence while sympatric or allopatric to its kin species, the blunt‐snout bream, aiming to reveal the population genetic differentiation characteristics, elucidating possible driving factors that promote these differentiations, and giving hints for future conservation.

## 2 Materials and Methods

### 2.1 Ethics Statement

All study procedures and collection of samples were approved by the Animal Experimental Ethical Inspection Form of Institute of Hydrobiology, Chinese Academy of Sciences (approval number: IHB2024‐601).

### 2.2 Sampling and Genome Sequencing

In September 2022, one wild black bream specimen was collected from Poyang Lake, Xingzi, China, for genome sequencing. To facilitate genome annotation, total RNAs from tissue samples, including muscle, gill, kidney, liver, heart, and brain, were promptly excised, preserved in liquid nitrogen, and then stored at −80°C. High‐quality genomic DNA was extracted from the muscle tissue using the FastPure Cell/Tissue DNA Isolation Mini Kit (Vazyme, DC102‐01) in accordance with the manufacturer's protocol.

For the initial genome survey, paired‐end libraries with an insert size of 400 bp were constructed and sequenced using the MGISEQ‐T7 platform with read lengths of 150 bp. To support the genome assembly process, 15 kb PacBio libraries were generated and sequenced under CCS (circular consensus sequencing) mode on the PacBio Sequel II system. Additionally, high‐throughput chromatin conformation capture (Hi‐C) libraries were prepared and sequenced on the MGISEQ‐T7 platform to facilitate chromosome‐level genome assembly.

For population genetic analysis, genomic DNA was extracted from the muscle tissues of 39 individuals across three populations. These included one black bream population from the Heilongjiang River (133°60′E, 48°13′N) with 11 individuals (MSnp) and another from Poyang Lake (116°3′E, 29°22′N) with 15 individuals (MSpl). Additionally, a population of *M. amblycephala* was also sampled from Poyang Lake (MA; *n* = 13).

### 2.3 Chromosome‐Level Assembly of the Black Bream Genome

To estimate the genome size and heterozygosity of the black bream, we analyzed the filtered sequencing data using a *k*‐mer size of 17 to assess the frequency distribution. The genome size (G) was calculated using the formula *G* = *Nk*‐mer/*Dk*‐mer, where *Nk*‐mer is the total count of *k*‐mers, and *Dk*‐mer is the average depth of 17‐mers.

The initial genome assembly was conducted using Hifiasm v0.13.0 (Cheng, Jarvis, et al. 2022) with default settings, followed by polishing with Nextpolish v1.3.3 (Hu, Fan, et al. 2020). For the chromosome‐level assembly of the genome, we employed the Juicer v1.9.9 (Durand et al. 2016) and 3D‐DNA v1.9 (Dudchenko et al. 2017) pipelines to cluster, order, and align contigs into chromosome‐level scaffolds. The integrity of the final genome assembly was assessed with the Benchmarking Universal Single‐Copy Orthologs (BUSCO) software v3.0.2 (Simao et al. 2015), using the “actinopterygii_odb9” library for evaluation. The genome collinearity between the assembled black bream genome and that of its kin species was evaluated through pairwise alignments, sequence identity calculations, and plotting conserved synteny relationships using Jcvi v1.3.9 (Tang et al. 2008), with a minimum span threshold of 30 genes for synteny blocks.

### 2.4 Genome Annotation

To annotate transposable elements (TEs) and tandem repeats within the black bream chromosome‐level genome, a dual‐faceted approach was employed. Initially, known repeats were identified utilizing a homology‐based prediction technique with RepeatMasker v4.1.2 (Tarailo‐Graovac and Chen 2009) and RepeatProteinMask v4.0.8 (Bao, Kojima, and Kohany 2015) against the RepBase TE library (https://www.girinst.org/repbase/). Following this, we used a de novo strategy to identify novel repetitive elements. We employed RepeatModeler v1.0.8 (Tarailo‐Graovac and Chen 2009) to create a custom repeat database. This database was then used with RepeatMasker to annotate transposable elements (TEs). Tandem repeats were annotated with Tandem Repeat Finder v4.10.0 (Benson 1999).

The structural annotation of protein‐coding genes within the black bream genome was conducted after soft‐masking of all repeat sequences, employing a comprehensive strategy that integrates ab initio prediction, homology‐based analysis, and RNA‐seq data. For homology‐based prediction, protein sequences from five species— *Ancherythroculter nigrocauda* , *Ctenopharyngodon idella* , *Cyprinus carpio, Danio rerio, and Megalobrama amblycephala*—were aligned to the black bream genome, with homologous genes predicted using MiniProt v0.12 (Li 2023). For de novo prediction, we used tools such as Augustus v3.2.3 (Stanke et al. 2008), GeneID v1.4 (Blanco, Parra, and GUIGó 2007), GlimmerHMM v3.0.4 (Majoros, Pertea, and Salzberg 2004), and SNAP v6.0 (Lomsadze et al. 2005). Transcriptome‐based annotation involved mapping RNA‐seq data onto the black bream genome with HISAT2 v2.1.0 (Kim et al. 2019) and assembling it using StringTie v1.3.5 (Kovaka et al. 2019). Open reading frames (ORFs) were predicted with TransDecoder v5.5.0 (https://github.com/TransDecoder/TransDecoder). Finally, a comprehensive gene set was generated by EvidenceModeler v1.1.1 (Haas, Salzberg, and Zhu 2008) and annotated for protein‐coding gene structure using PASA v2.4.1 (Haas et al. 2003).

Gene functional annotation was conducted using Blast+ v2.29 (McGinnis and Madden 2004) with an *E*‐value threshold of 1E‐5 across five public protein databases: the Kyoto Encyclopedia of Genes and Genomes (KEGG), Gene Ontology (GO), EuKaryotic Orthologous Groups (KOG), SwissProt, and the NCBI non‐redundant (NR) protein sequence database. Additionally, protein domains and motifs were annotated with InterProScan (Mulder and Apweiler 2007).

For annotating noncoding RNAs (ncRNAs) within the black bream genome, RNAmmer v1.2 (Karin et al. 2007) was deployed for ribosomal RNA (rRNA) prediction, while tRNAScan‐SE v2.0 (Lowe and Eddy 2019) facilitated the identification of transfer RNA (tRNA). Furthermore, the detection of other ncRNAs was accomplished through alignment against the Rfam database.

### 2.5 Gene Family Identification and Phylogenomic Analysis

To explore the evolutionary dynamics of gene families in the black bream genome, OrthoFinder v2.5.2 (Emms and Kelly 2019) was utilized to identify orthologous groups across 11 species, including *Danio rerio* , *Onychostoma macrolepis, Cyprinus carpio, Ctenopharyngodon idella, Hypophthalmichthys molitrix, Hypophthalmichthys nobilis, Ancherythroculter nigrocauda, Culter alburnus, Parabramis pekinensis, M. amblycephala, and M. skolkovii*. These species were selected because they include kin species, closely related species (same subfamily), or the same tribe species of the black bream. We also added some model species, such as zebrafish, etc., which can provide a good background for the evolutionary analysis of black bream genomic structure. The identified single‐copy orthologous genes were utilized for the construction of the phylogenetic tree and the estimation of divergence times. Using MAFFT v7.471 (Katoh and Standley 2013) for sequence alignment, we subsequently removed poorly aligned regions with Gblocks v0.91b (Castresana 2000); phylogenetic relationships were then deduced via IQ‐TREE2 v2.2.5 (Lam‐Tung et al. 2015), with zebrafish serving as the outgroup. Divergence times were estimated through MCMCTREE within the PAML package (Yang 2016), incorporating three calibration points from the TimeTree (Kumar et al. 2017) database (*C. carpio*‐*O. macrolepis*: 13.0–68.2 MYA; *H. molitrix*‐*H. nobilis*: 3.5–10.8 MYA; *A. nigrocauda*‐*C. alburnus*: 1.69–6.62 MYA). The expansion and contraction of gene families were analyzed using CAFÉ v4.2 (De Bie et al. 2006). Furthermore, GO and KEGG enrichment analyses were conducted on expanded and contracted gene families via the clusterProfiler package in R v4.3.3 (R Core Team 2011).

### 2.6 Whole‐Genome Resequencing and SNP Calling

Genomic DNA from 39 samples (MSnp, *n* = 11; MSpl, *n* = 15; MA, *n* = 13) was used to generate libraries with an average insert size of 300 bp, which were then sequenced on the MGISEQ‐T7 platform. Before mapping reads, we utilized fastp v0.23.3 (Chen et al. 2018) to remove adapter sequences and trim low‐quality bases using default parameters. The clean data were mapped to the soft‐masked black bream genome using BWA‐MEM v0.7.17 (Li and Durbin 2009) with default settings. The resulting SAM files were converted to BAM format and sorted using SAMtools v1.9 (Li et al. 2009). PCR duplicates were then identified and removed using Picard v 2.25.1 (https://broadinstitute.github.io/picard/) MarkDuplicates tool. We employed GATK v4.1 (Mckenna et al. 2010) for variant calling, employing the HaplotypeCaller method to generate individual‐specific gVCF files. Subsequently, these individual gVCF files were aggregated using the CombineGVCFs method, resulting in a single gVCF file. This gVCF file was then transformed into a VCF file using the GenotypeGVCFs method. To obtain high‐quality SNPs for downstream analysis, variants were removed using VariantFiltration in GATK with the parameters ‘‐‐filter‐expression QD < 2.0 || MQ < 40.0 || FS > 60.0 || SOR > 3.0 || MQRankSum < −12.5 || ReadPosRankSum < −8.0’. We further filtered the genomic variants for population analysis using VCFtools v0.1.16 (Danecek et al. 2011), applying the parameters ‘‐‐remove‐indels ‐‐min‐alleles 2 ‐‐max‐alleles 2 ‐‐minDP 10 ‐‐maf 0.05 ‐‐max‐missing 0.8’.

### 2.7 Population Genetic Analysis

For the population genetic analysis of black bream, we inferred phylogenetic relationships using SNP data and constructed a maximum‐likelihood tree with IQ‐TREE v2 (Lam‐Tung et al. 2015), using a GTR + ASC substitution model and 1000 bootstrap replicates. We filtered SNPs for linkage disequilibrium (LD) using PLINK v1.9 (Chang et al. 2015) with the settings “‐‐indep‐pairwise 50 10 0.5,” resulting in 1,771,258 SNPs for further analysis. Principal component analysis (PCA) was performed with the same software, and PCA plots were created using the “ggplot2” package in R. Population structure was inferred with ADMIXTURE v1.3.0 (Alexander and Lange 2011), setting ancestry components (*K*) values from 1 to 5. Sequentially Markovian Coalescent (SMC++) v1.15.4 (Terhorst, Kamm, and Song 2016) was employed to simulate the demographic history of the two populations, allowing us to infer historical changes in their effective population sizes and divergence time. For this analysis, we selected the five individuals with the highest sequencing depth from each population. Initially, we used vcf2smc to convert VCF files into the required SMC++ format. Following this, the estimate command was applied to fit a detailed population size history to the data. Additionally, we used the split command to model clean split events between the two populations, utilizing marginal estimates from the estimate results. This analysis was conducted with a mutation rate of 1 × 10^−9^ per year and an assumed generational span of 2.5 years (Chen et al. 2022).

### 2.8 Genome‐Wide Patterns of Genetic Diversity and Linkage Disequilibrium Analysis

We utilized VCFtools to analyze genetic diversity and differentiation within and among populations. This involved calculating the average nucleotide diversity (π), Tajima's *D*, and both global and pairwise *F*st statistics over a 25 kb sliding window, using parameters “‐maf 0.05 ‐max‐missing 0.8 ‐minDP 10”. To delineate the genome‐wide linkage disequilibrium (LD) decay across three populations, we computed the average *r* ^2^ values for marker pairs exhibiting a minor allele frequency (MAF) above 0.05, utilizing PopLDdecay v3.4 (Zhang et al. 2019) with a cap of 500 Kb for the maximal inter‐SNP distance. Each population was analyzed independently, following the extraction of SNPs specific to it for this assessment.

### 2.9 Four‐Taxon *D*‐Statistic Test

Utilizing genome‐wide SNP allele frequency data, ABBA‐BABA tests are crucial for discerning levels and patterns of admixture across phylogenetic branches in populations or closely related species. In our study, we utilized the Dtrios function in the Dsuite package v0.5 (Malinsky, Matschiner, and Svardal 2021) to estimate classic 4‐taxon *D*‐statistics. The populations included MSpl, MSnp, and MA, with *Parabramis pekinensis* serving as the outgroup (detailed information is shown in Table A7). We set the number of jackknife blocks to 20 and specified a jackknife block size of 1000. This approach, framed within the phylogenetic structure {[(P1, P2), P3], O}, facilitates the identification of differential admixture between P3 and P1 (BABAs) or P2 (ABBAs), based on shared derived alleles. This method provides insights into the genetic relationships and admixture dynamics among these taxa.

### 2.10 QuIBL Analysis

In this study, we utilized QuIBL (Edelman et al. 2019), a method based on internal branch length distribution, to assess the contributions of ILS and introgression to distinguish between models incorporating both ILS and introgression and those with ILS only, providing localized insights into introgression patterns. For our analysis, we selected one sample with the highest coverage per species (MSpl10 represents the MSpl population, MSnp61 represents the MSnp population, and MA13 represents the MA population), with *Parabramis pekinensis* serving as the outgroup. Given QuIBL is sensitive to recombination, we employed Seqkit (Shen et al. 2016) to extract small 10‐kb windows separated by 100 kb from each sample, reducing the likelihood of including a recombination breakpoint. We then filtered out windows containing 50% or more missing data. Subsequently, we utilized a custom Python script to generate sliding window trees for the resulting 10,460 windows. Each alignment window was subjected to tree inference using IQ‐TREE. Finally, the resulting 10,460 trees were then utilized as input for QuIBL to conduct further analysis.

### 2.11 Delineation of Genomic Regions Under Selection

Genomic selective sweep analysis was conducted to identify candidate regions and genes under selection in contrasting northern and Poyang Lake ecological environments, underlying intraspecific variation in black bream populations. We utilized VCFtools for the estimation of π ratio and *F*st across 25‐kb nonoverlapping sliding windows. We identified genomic regions as selective sweep areas in the MSnp population where both the top 1% values for *F*st and the top 5% values for π ratio overlapped. Additionally, regions simultaneously meeting the top 1% criteria for *F*st values and the bottom 5% criteria for π ratio were identified as selective areas in the MSpl population. The genes located within or overlapping the sweep regions were earmarked for further gene GO and KEGG pathway enrichment analyses.

## 3 Results

### 3.1 Chromosome‐Scale Genome Assembly of Black Bream

By integrating MGI short reads, PacBio HiFi reads, and Hi‐C reads, we successfully constructed a chromosome‐level, high‐quality genome assembly for the black bream (Table A1). After filtering, 70.40 Gb of clean MGISEQ data were retained to perform genome survey. Our survey analysis revealed a heterozygosity rate of approximately 0.45% and a repetitive sequence composition constituting 62.77% of the genome (Table 1).

**TABLE 1:** The result of *k*‐mer analysis.

*K*‐mer	*N K*mer	Peak depth	Genome size/bp	Heterozygous rate/%	Repeat rate/%
17	52,337,591,904	48	1,054,560,000	0.450	62.77

**FIGURE 3:**
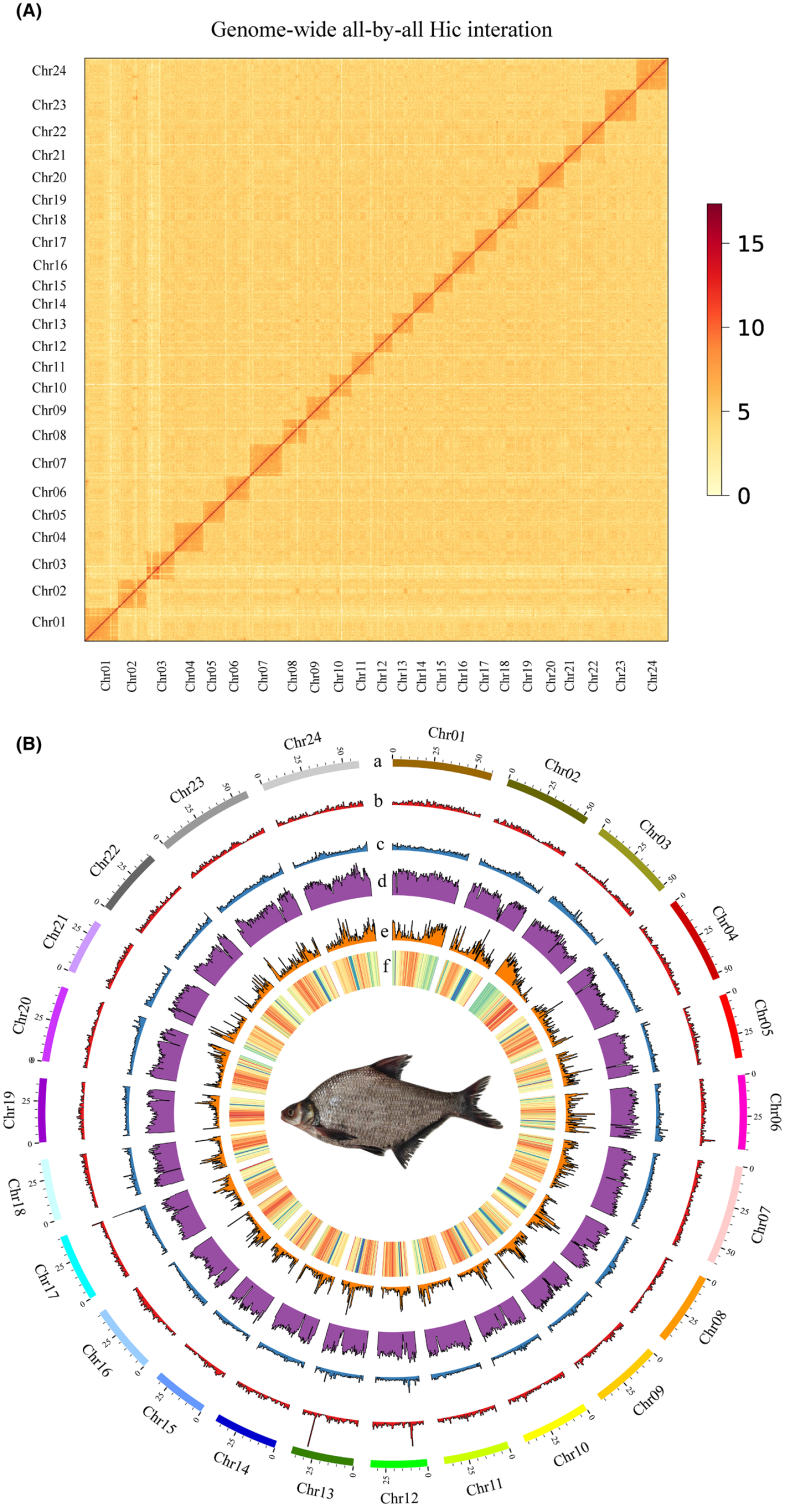
Characteristics of the black bream genome. (A) Interaction matrix across the black bream genome; blocks with higher color intensity indicate stronger contacts. (B) Circos graph (from outside to inside) representing (a) 24 chromosomes of the genome. (b) Gene density. (c) GC Density. (d) Distribution of DNA transposons (DNA). (e) Distribution of long terminal repeats (LTR). (f) Distribution of transposable elements (TEs).

HiFi reads from PacBio sequencing finally culminated in an assembly size of 1.13 Gb across 200 contigs, with a significant contig N50 size of 25.92 Mb. This genome size was similar to the length estimated from the *k*‐mer analysis (Table 1). The GC content of the genome is approximately 37.78%. Following assembly, Hi‐C data facilitated the anchoring and orientation of sequences across 24 chromosomes (Figure 3A), with chromosome lengths varying between 33.51 and 60.53 Mb, as detailed in Table 2. This arrangement accounted for approximately 94.95% of the total genome coverage. The final rendition of the black bream genome comprised 96 scaffolds, achieving an aggregate length of 1,133,966,472 bp, and was characterized by a scaffold N50 of 42.08 Mb (Table A2).

**TABLE 2:** Distribution of chromosome lengths in black bream.

Chr	Length	Scaf num
Chr01	60,537,602	5
Chr02	60,310,966	5
Chr03	59,022,035	3
Chr04	53,234,652	3
Chr05	39,991,036	5
Chr06	46,315,395	5
Chr07	59,852,415	4
Chr08	41,808,494	2
Chr09	42,083,675	3
Chr10	38,870,996	1
Chr11	41,091,564	1
Chr12	41,138,067	3
Chr13	41,436,427	3
Chr14	51,669,952	5
Chr15	33,913,182	5
Chr16	45,380,377	3
Chr17	53,093,155	6
Chr18	39,614,504	4
Chr19	44,553,544	1
Chr20	38,142,820	2
Chr21	34,426,610	3
Chr22	33,516,584	1
Chr23	39,054,048	3
Chr24	37,603,023	2
Total	1,016,123,521	78

### 3.2 Genome Annotation

Our analysis identified a total of 674.30 Mb of repetitive sequences, representing 59.51% of the whole genome. Among these, DNA transposons (30.55%) and long terminal repeat retrotransposons (9.06%) were the dominant transposable elements (Figure 3B and Table A3).

**FIGURE A1:**
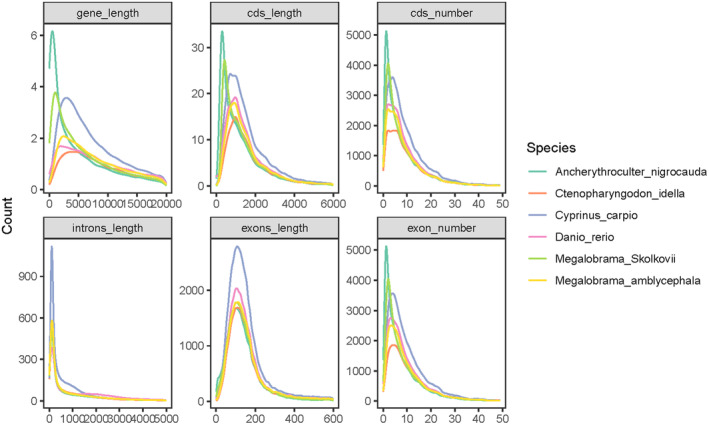
A comparison of gene structure features between black bream and five other cyprinid fish species is presented. The data include the lengths of coding sequences, exons, and introns, along with the exon numbers of genes.

Employing a comprehensive genomic annotation approach that combined ab initio predictions, homology‐based analyses, and RNA‐seq methodologies, we uncovered a total of 32,698 protein‐coding genes. Comparative analysis demonstrated that our predicted gene models exhibited distribution patterns like those of five other cyprinid fish species ( *Ancherythroculter nigrocauda* , *Ctenopharyngodon idella* , *Cyprinus carpio, Danio rerio, Megalobrama amblycephala*) in terms of the number and length of coding sequences, exons, and introns (Figure A1 and Table A4).

Furthermore, functional annotations based on the GO, KEGG, KOG, SwissProt, and NR databases were successfully assigned to 30,558 genes, representing 93.46% of the total genes (Table A5). To assess the completeness of the genome, BUSCO analysis yielded integrity scores of 98.5% and 96% for the assembled genome and gene set, respectively (Table A6). These results collectively affirmed the high quality of the black bream with chromosome‐level genome assembly.

### 3.3 Comparative Genomic and Evolutionary Analyses

**FIGURE 4:**
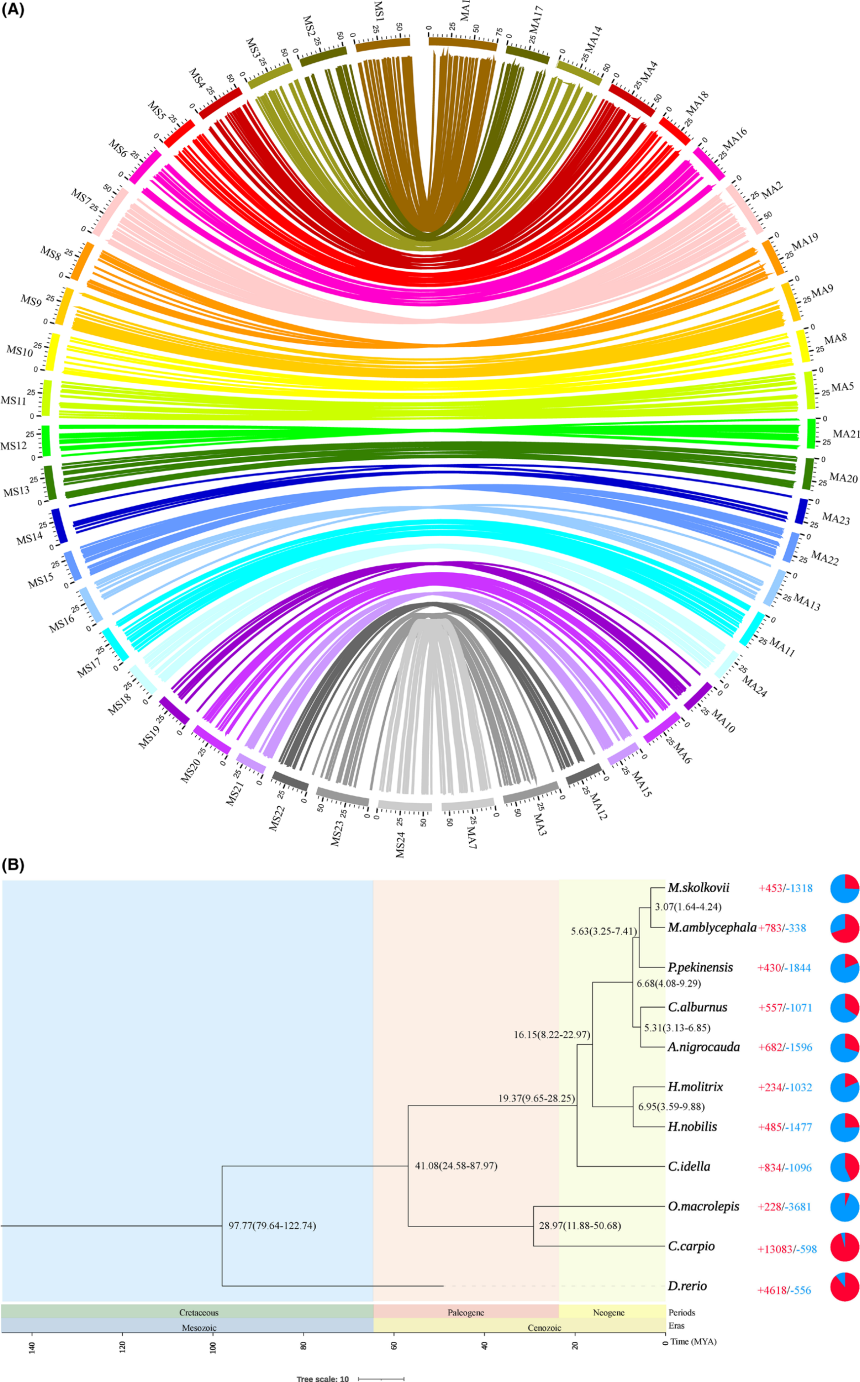
Synteny and phylogenetic analysis of black bream and other representative species. (A) Circos diagrams showing black bream (left) chromosome synteny relations with *M. amblycephala* (right). The lines between the genomes indicate syntenic blocks. (B) The numbers near the ancestral nodes indicated the estimated divergence time (MYA), with the 95% confidence intervals in parentheses. Expansion and contraction of gene families were indicated by blue and red markers (represented by numerical values and circle symbols), respectively.

The collinearity of the black bream genome was assessed by constructing synteny blocks using the *M. amblycephala* genome. Genome collinearity analysis revealed a one‐to‐one alignment among the 24 chromosomes of the two genomes, identifying a total of 238 syntenic blocks, each spanning at least 30 genes (Figure 4A). This high degree of concordance suggests a close evolutionary relationship between these kin species. Additionally, our phylogenetic analysis, based on 2207 single‐copy orthologs, indicated that the divergence between black bream and its kin species *M. amblycephala* occurred approximately 3.07 million years ago (MYA).

Gene family evolutionary analysis demonstrated that the black bream genome underwent expansion in 453 gene families and contraction in 1318 gene families compared to its most recent common ancestor (Figure 4B). Functional enrichment analysis of the expanded gene families revealed significant enrichment across 101 GO terms and 11 KEGG pathways (Data S1), primarily associated with mitochondrial fission, GTP binding, and viral response. Enriched pathways such as ErbB, GnRH, and Wnt signaling, neuroactive ligand‐receptor interaction, and progesterone‐mediated oocyte maturation point to key processes in neural regulation, energy metabolism, immune response, and reproductive adaptation. These biological characteristics likely enhance the species' ecological adaptability to diverse aquatic environments.

### 3.4 Population Genetic Diversity and Linkage Disequilibrium

**FIGURE 5:**
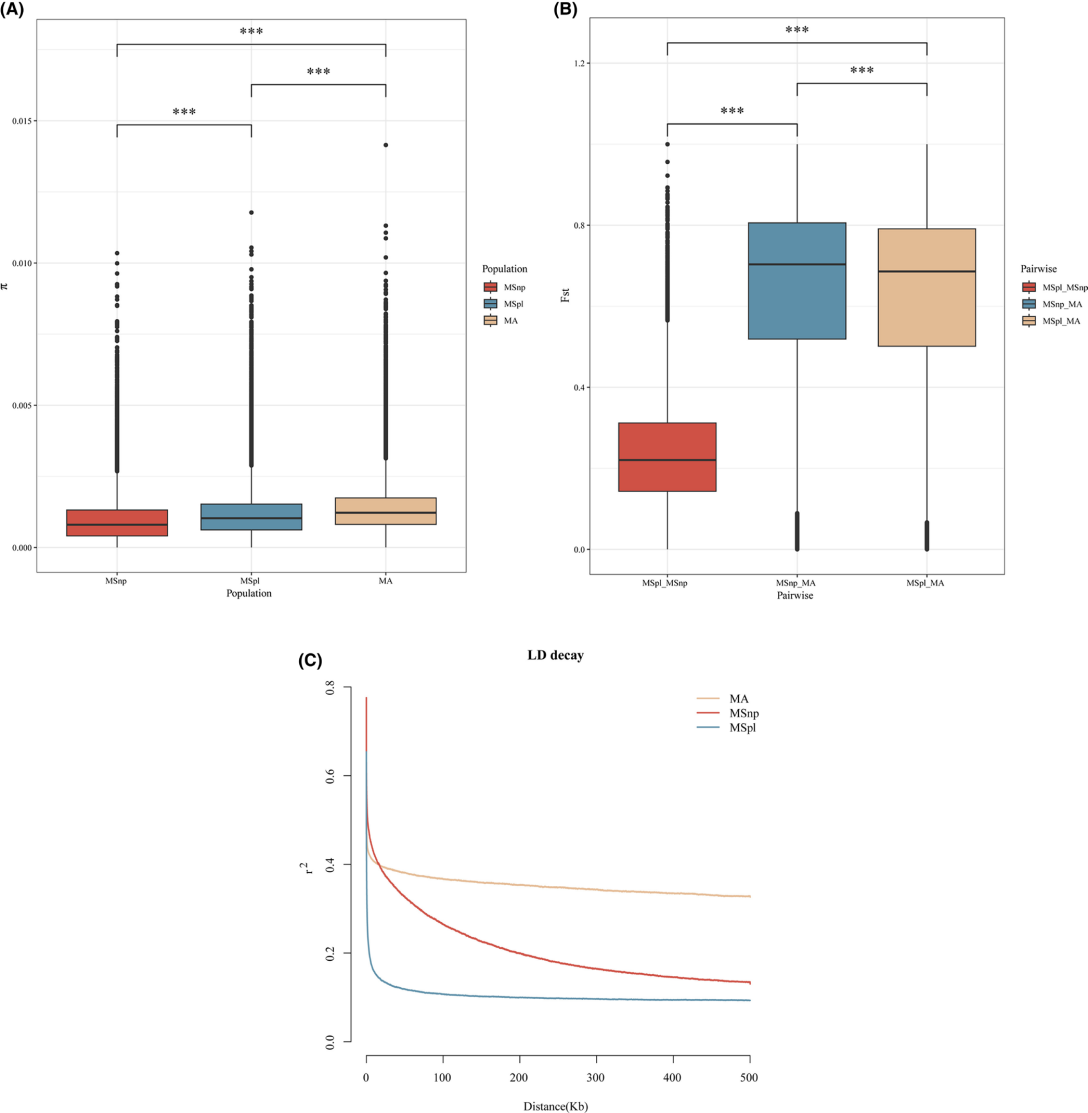
Patterns of genome‐wide divergence between black bream and *M. amblycephala* in 25‐kb non‐overlapping windows. (A) Nucleotide diversity (π) in three populations (Asterisks (***) indicate *t* test *p* < 2.2e‐16). (B) Pairwise *F*st values between three different population comparisons: MSpl vs. MSnp, MSnp vs. MA, and MSpl vs. MA (Asterisks (***) indicate *t* test *p* < 2.2e‐16). (C) Linkage disequilibrium analysis.

We resequenced 39 samples from the Heilongjiang River (northern population, MSnp) and Poyang Lake (MSpl). An average size of 26.19 Gb of data per individual was produced, yielding an average coverage and mapping rate of 22.85× and 98.65%, respectively (Table A7). A total of 12,556,656 high‐quality SNPs were identified after SNP calling and filtering. To assess the genetic variability and differentiation between the northern and Poyang Lake populations of black bream, we estimated the nucleotide diversity (π) and Tajima's *D*, along with the pairwise population differentiation coefficient *F*st. The MSpl population showed slightly higher genetic diversity compared to the MSnp population (π: 1.19 × 10^−3^ vs. 0.97 × 10^−3^; Tajima's *D*: 0.834 vs. 0.472; Figure 5A and Table A8). Further analysis of *F*st revealed that the genetic differentiation between the MSnp population and the MA ( *M. amblycephala* ) population was slightly greater than that between the MSpl population and the MA population (*F*st: 0.638 vs. 0.621; *t*‐test *p* < 2.2e‐16; Figure 5B).

Significant variation was observed in the LD decay curves among black bream populations. The MSpl population displayed a steep decline in LD within the first 50 Kb, with *r* ^2^ rapidly falling below 0.2, indicating a historically higher recombination rate or greater genetic diversity than the MSnp population. The MSnp population demonstrated a more gradual LD reduction, retaining a higher level of LD over the same genetic distance (Figure 5C), implying a more pronounced founder or bottleneck effect. These patterns may be indicative of reduced genetic variability in the MSnp population.

### 3.5 Population Structure Analyses

**FIGURE 6:**
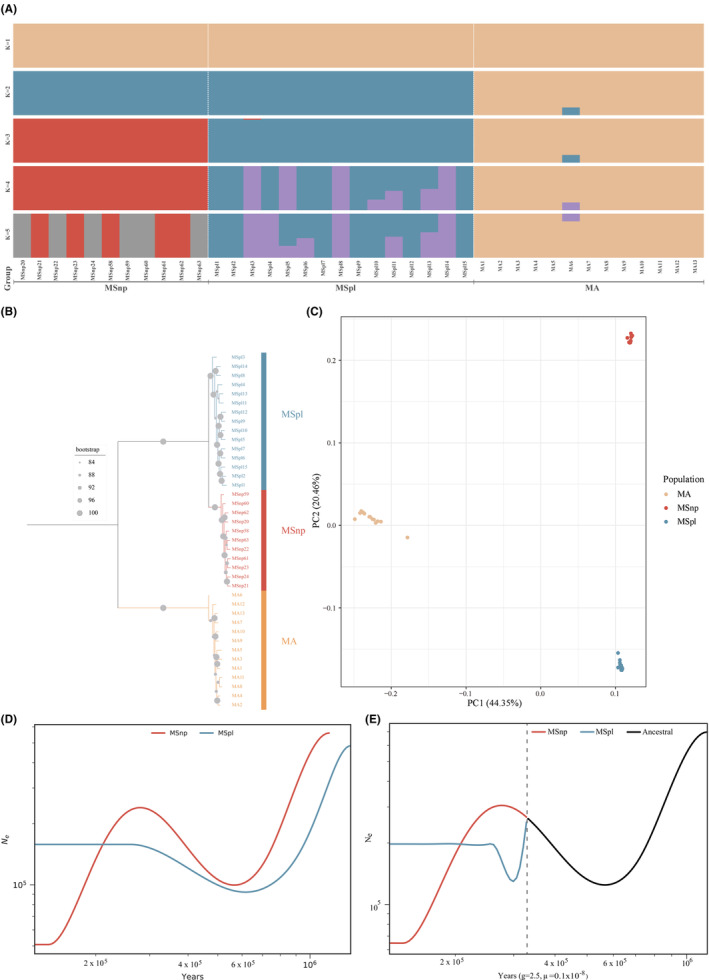
Population structure analysis and demographic history of *M. amblycephala* and black bream populations. (A) Genetic structure with different ancestry components (*K* = 1–5). Each bar represents an individual, and different colors represent the proportion contributed by that ancestral population. (B) Maximum‐likelihood tree derived from whole‐genome SNPs, with branches for the Poyang Lake and Heilongjiang populations of black bream indicated in blue and red, respectively; the *M. amblycephala* population is highlighted in yellow. The scale bar represents the genetic distance between individuals. (C) PCA plots of the first two components of the 39 individuals. (D) Demographic history of MSnp (red) and MSpl (blue) inferred from SMC++ analysis. Both populations showed significant decline. (E) SMC++ analysis of divergence time between MSnp and MSpl populations. The black line represents the ancestral population. The gray dashed line marks the estimated divergence time at approximately 0.32 MYA.

We further analyzed the phylogenetic relationships and population structure of the northern and Poyang Lake black bream populations using the resequencing data. Population structure analysis revealed distinct genetic clusters. At the best‐supported structure, where *K* = 3, the black bream further divided into two groups, aligning with their geographic locations as shown in Figure 6A. As anticipated, both the phylogenetic tree and PCA corroborated the results of the admixture analysis. The phylogenetic tree confirmed clear genetic divergence between the MSpl and MSnp populations (Figure 6B). In the PCA, the primary eigenvector, which accounted for 44.35% of the total genetic variance, also differentiated the black bream from *M. amblycephala* . The secondary eigenvector further distinguished between the black bream populations, explaining 20.46% of the genetic variance (Figure 6C).

The SMC++ analysis revealed a significant decline in effective population size for both populations during the period of 2 ‐ 0.6 MYA (Figure 6D). Divergence between the MSnp and MSpl populations was estimated to have occurred around 0.32 MYA (Figure 6E). Following this split, the northern population experienced an additional sharp reduction in effective population size approximately 0.29 MYA, reflecting further demographic fluctuations.

### 3.6 Genetic Differentiations of Black Bream Populations Allopatric or Sympatric to *M. amblycephala*

**FIGURE 7:**
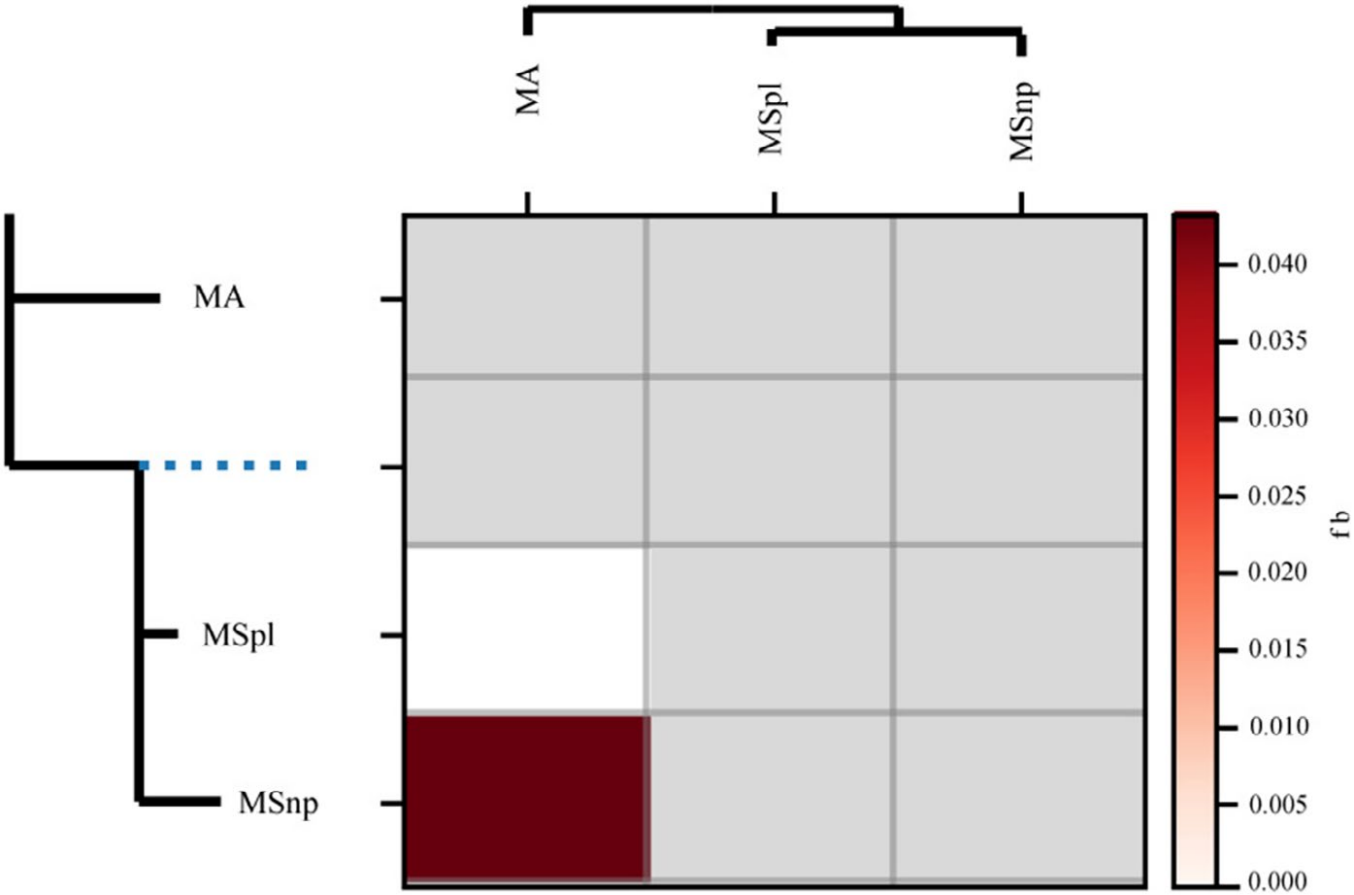
ABBA‐BABA statistics of introgression. Introgression proportions inferred from interspecies pairs undergoing introgression are mapped to internal branches using the f‐branch approach. The extended tree to the left of each matrix displays both terminal and ancestral branches.

We conducted *D*‐Statistics to further identify the genetic differences in black bream populations that are either sympatric or allopatric to MA. We used an absolute *Z*‐value greater than 3 as the criterion to reject the null hypothesis, corresponding to a *p*‐value of less than 0.002. Our ABBA‐BABA tests unveiled a genome‐wide abundance of allele sharing between the MSnp and MA populations (Figure 7), suggesting that interspecific gene flow in allopatric populations (between P2 and P3) was greater than that between sympatric populations (between P1 and P3) (Table A9).

In addition, we utilized QuIBL to assess the likelihood that the internal branch length distribution aligns with either the model featuring introgression and ILS or with ILS alone among the MSnp, MSpl, and MA populations, using *P. pekinensis* as an outgroup. After filtering based on a significance determination value of |dBIC| > 10, our analysis revealed that approximately 8% of loci support introgression under the scenario of ILS, and 59% of loci suggest ILS (Data S2).

### 3.7 Selection Signals in Northern and Poyang Lake Populations of Black Bream

**FIGURE 8:**
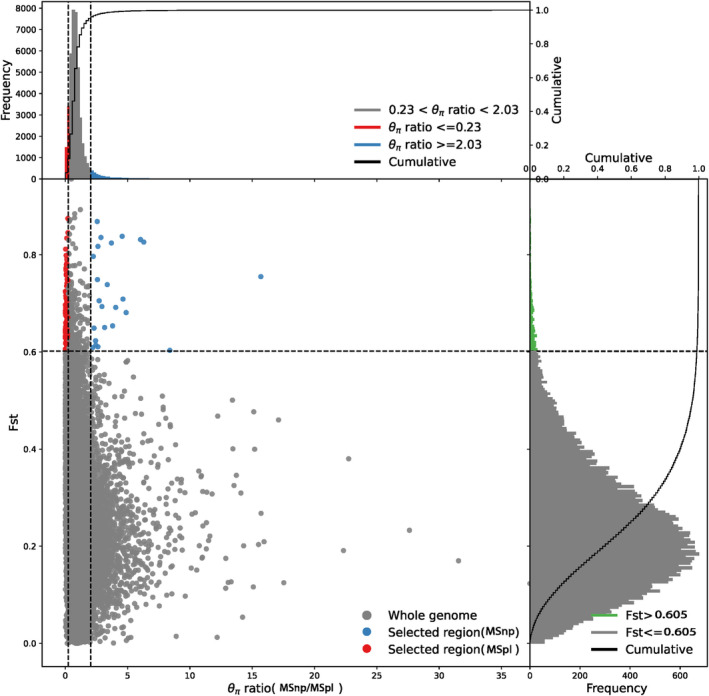
Identification of divergent regions between MSnp and MSpl populations. The distribution of the π ratio (MSnp/MSpl) and *F*st values was calculated across 25 kb non‐overlapping windows. Blue dots indicate the selective sweep regions of the MSnp population (top 5% π ratio ≥ 2.03 and top 1% *F*st ≥ 0.605). Red dots indicate the selective sweep regions of the MSpl population (bottom 5% π ratio < 0.23 and top 1% *F*st ≥ 0.605).

We employed a combination of *F*st and π analyses between the northern and Poyang Lake populations of black bream to investigate the selection signals driving the genetic differentiation between these two populations. We identified selection acting on 22 genes within 31 genomic regions in the MSnp population and 159 genes within 217 genomic regions in the MSpl population (Figure 8). GO and KEGG analyses revealed that these genes were highly enriched in sensory perception of chemical stimulus, pineal gland development, eye morphogenesis and inner ear development, fin regeneration, G protein‐coupled receptor signaling pathway, ATPase activity, skeletal muscle fiber development, positive regulation of Wnt signaling pathway, and motor neuron migration (Data S3).

## 4 Discussion

### 4.1 Characteristics of the Black Bream Genome

In the present study, the final assembled genome size of the black bream was 1.13 Gb, marginally surpassing that of its kin species *M. amblycephala* (Liu et al. 2021). Furthermore, the genome size of black bream also exceeded those of its relatives, the four major Chinese carps, silver carp (816 Mb), grass carp (871 Mb), black carp (853 Mb), and bighead carp (868 Mb) (Wang et al. 2024). In comparison to the genome assemblies of other species, the black bream assembly distinctly benefited from an enhanced contig N50 length of 25.92 Mb, surpassing that of *Culter alburnus* , which had a contig N50 of 17.8 Mb (Jiang et al. 2023), significantly outstripping the 3.12 Mb contig N50 length reported for *Ancherythroculter nigrocauda* (Zhang et al. 2020), and exceeding the contig N50 length of *M. amblycephala* (2.6 Mb).

Black bream, like its kin species, possessed 24 chromosomes, and its genome encompassed 32,698 predicted genes, slightly surpassing the 30,357 genes identified in *M. amblycephala* . Furthermore, the high degree of genome collinearity observed between black bream and *M. amblycephala* indicated a close evolutionary connection between these kin species. Phylogenetic analysis revealed that black bream and *M. amblycephala* diverged approximately 3.07 MYA, coinciding with the late Pliocene's global cooling.

The divergence of the adhesive‐egg‐laying Cultrinae species and the drifting‐egg‐laying grass carp *C. idellus* occurred 15.92 MYA during the middle Miocene, which is in agreement with previous findings (Clift et al. 2008; Jiang et al. 2023), being coincident with the intensified activity of the Asian monsoon in that period (He et al. 2004; Cheng, Yu, et al. 2022). Analysis of gene family evolution revealed that the black bream genome experienced expansion in 447 gene families and contraction in 1358 gene families relative to its most recent common ancestor. The functional enrichment of gene families underlines the significance of immune response, xenobiotic metabolism, and energy conversion processes in supporting the black bream's lifestyle in diverse and potentially challenging aquatic environments.

### 4.2 Contrasting Geographic Population Genetic Differentiations

In the present study, a slightly higher *F*st value was found between MSnp and MA populations than that between MSpl and MA populations. This indicated that the black bream northern population likely has a more pronounced genetic differentiation.

It has been widely found that in allopatric populations, bottlenecks, sometimes severe and prolonged, may occur, leading to a reduction in genetic diversity due to the increased genetic drift and diminished effectiveness of natural selection (Hunter et al. 2010; Pastor et al. 2004; Roques and Negro 2005; Jangjoo et al. 2016; Weber, Stewart, and Lehman 2004). In this study, the black bream population from Poyang Lake exhibited higher genetic diversity, elevated Tajima's *D* values, and lower levels of LD, suggesting it represents the ancestral population. For the northern black bream population, the allopatric distribution and the lower genetic diversity showed that its northern distribution was later dispersion, and bottlenecks must have occurred after its colonization.

Bottlenecks may also cause slower linkage disequilibrium decay compared to native populations, in addition to the severe reduction in genetic diversity following colonization (Chen et al. 2021). Therefore, reconstruction of ancestral demographic history can reveal bottlenecks during founder events (Foote et al. 2016). In the present study, increased linkage disequilibrium and slower decay were found in the black bream northern population. The two sharp declines in demographic analysis indicated that it possibly experienced two severe population bottlenecks.

As evolutionary histories occur over long timescales, it is possible that more complex processes or contributing factors, such as range expansion followed by fragmentation or the loss of ephemeral dispersal routes driven by eco‐geographic changes, could contribute to the observed patterns. At present, distinguishing between founder effects, bottlenecks, and range expansion‐fragmentation dynamics can be challenging, particularly when relying solely on summary statistics. We hope that, with additional data and more comprehensive demographic modeling in future analyses, further insights will be gained to better understand the population history.

### 4.3 Retention of Ancestral Polymorphisms in the Allopatric Population

Given the lower genetic diversity and more pronounced genetic differentiation, it is natural to anticipate that the black bream northern population should have more novel components. However, our ABBA‐BABA tests revealed that the black bream northern population had more shared polymorphisms with *M. amblycephala* than the Poyang Lake ones. This indicated that the black bream northern population retained more ancestral alleles. The sharing of unique alleles between one population and its related relatives is usually caused by ILS or hybrid introgression, which is the major reason causing the conflicts between species trees and gene trees.

Incomplete lineage sorting is common across organisms because allelic polymorphisms often persist through multiple speciation events (Mao et al. 2021; Meleshko et al. 2021), leading to the generation of shared polymorphisms among closely related species, which may play crucial roles in processes of adaptation and speciation (Hobolth et al. 2011). Since the black bream northern population and *M. amblycephala* population are allopatric, contemporary introgression is impossible. Therefore, the above phenomena should be caused by ILS or ancient introgression. Our QuIBL analysis suggested that approximately 59% of loci support ILS, with low levels of post‐speciation interspecific gene exchange at 8%. Additionally, the lack of recent admixture signs in the ADMIXTURE analysis suggested that current interspecific introgression is limited due to strong reproductive isolation between the populations. The inferred introgression likely occurred early in the diversification process, primarily among the ancestral lineages (Meleshko et al. 2021).

The lower genetic diversity and sharing of ancestral alleles in the black bream northern population were possibly related to some local adaptations. For example, the black bream northern population needs at least 5 years to reach sexual maturity (Novomodny, Sharov, and Zolotukhin 2004), which could be driven by the prolonged isolation in unique habitats, demanding larger body size and higher intramuscular fat content (Hu et al. 2019) in cold water temperature. In contrast, it needs only 2–3 years to reach maturity in the southern population (Huang et al. 1997).

### 4.4 Genomic Basis for Population Differentiation

In this study, we identified some key genes related to reproductive processes, body size development, and muscle metabolism in the northern population. Black bream in the Heilongjiang River require at least 5 years to reach sexual maturity (Novomodny, Sharov, and Zolotukhin 2004). The prolonged segregation in unique habitats may promote the evolution of key genes essential for the breeding processes of this population. For example, the SOX2 gene, identified in selected regions of the MSnp population, is crucial for gametogenesis (Jiang et al. 2018). It plays a key role in the regulatory networks that maintain and proliferate spermatogonial stem cells (Patra et al. 2015) and support ovarian development (Yu et al. 2018), potentially contributing to the reproduction of this population. DNA polymerase alpha 1 (POLA1) has been identified as a crucial gene involved in the proliferation of fish myoblasts (Kong et al. 2021); this gene may be related to the larger body size observed in northern populations (Hu et al. 2019), potentially contributing to their growth and muscle development. SLC5A12, involved in the transport of lactate and pyruvate and playing a critical role in metabolic processes and energy production (Annaert et al. 2007), may contribute to the high intramuscular fat content observed in northern fish populations (Hu et al. 2019).

We further detected specific genes related to immune response, energy metabolism, and lipid metabolism in the black bream Poyang Lake population. This genetic profile likely reflected the competitive pressures faced by the Poyang Lake population, with dietary similarities and sympatric distribution with the MA population leading to ecological and nutritional competition. Consequently, the black bream Poyang Lake population may have required additional energy to evade competitors and effectively locate food resources.

## 5 Conclusions

This work presents a comprehensive chromosome‐level genome assembly for black bream and elucidates the genetic differentiation and local adaptation of its geographically distinct populations. Through whole genome re‐sequencing, we observed that the northern population exhibits reduced genetic diversity and higher genetic differentiation compared to the blunt‐snout bream, along with two significant declines in effective population size, suggesting historical bottlenecks due to allopatric isolation. Conversely, the Poyang Lake population maintains higher genetic diversity, indicative of ancestral characteristics. The northern population's greater retention of ancestral alleles is potentially due to incomplete lineage sorting and ancient introgression. Selection analysis highlighted key genes related to reproduction, body size, and muscle metabolism under selection in the northern population, likely contributing to its local adaptation. These insights into the genetic diversity and differentiation of black bream populations enhance our understanding of their evolutionary history and provide valuable information for conservation strategies.

## Author Contributions

**Ruijin Ding:** data curation (lead), formal analysis (lead), investigation (equal), methodology (lead), writing – original draft (lead), writing – review and editing (equal). **Dan Yu:** conceptualization (supporting), methodology (equal), validation (equal), writing – review and editing (equal). **Ke Yang:** methodology (supporting), resources (supporting). **Xinghua Wu:** methodology (supporting), resources (supporting). **Huanzhang Liu:** conceptualization (lead), investigation (equal), supervision (lead), validation (equal), writing – review and editing (equal).

## Conflicts of Interest

The authors declare no conflicts of interest.

## Supporting information


Data S1.

## Data Availability

All the sequencing data were deposited at the NCBI database and can be accessed under project ID PRJNA1127861. The analysis codes are available in Zenodo (https://doi.org/10.5281/zenodo.12543758).

## Appendix 1

**TABLE A1:** Statistics for the sequencing data of the black bream genome.

Library types	Sequencing platform	Insert size (bp)	Clean data (Gb)	Sequence coverage (x)
MGISEQ	MGISEQ‐T7RS	400	70.40	66.79
PacBio	PacBio SequeI II	15,000	28.66	25.30
HiC	MGISEQ‐T7RS	400	145.94	128.81

**TABLE A2:** The statistics of the assembled contigs and scaffolds for black bream genome.

StatType	ContigLength	ContigNumber	ScaffoldLength	ScaffoldNumber	GapLength	GapNumber
N50	25,630,223	17	42,083,675	11	500	61
N60	23,118,095	22	41,138,067	14	500	74
N70	16,864,464	27	39,614,504	17	500	86
N80	12,592,638	35	38,470,715	20	500	98
N90	7,238,702	47	34,426,610	23	500	110
Longest	44,553,544	1	60,537,602	1	500	122
Total	1,133,905,472	218	1,133,966,472	96	61,000	122
Length ≥ 1 kb	1,133,905,472	218	1,133,966,472	96	0	0
Length ≥ 2 kb	1,133,905,472	218	1,133,966,472	96	0	0
Length ≥ 5 kb	1,133,905,472	218	1,133,966,472	96	0	0

**TABLE A3:** Summary statistics of repetitive sequences annotation in black bream genome.

Type	Count	Length (bp)	Rate (%)
DNA	1,663,559	3,453,476	30.55
LINE	96,861	35,718,873	3.06
SINE	25,513	6,395,848	0.22
LTR	239,392	103,130,957	9.06
Other	497,129	31,823,192	2.63
Unknown	757,871	135,695,276	11.99
TandemRepeat	299,410	22,630,522	2.00
Total	3,280,325	316,217,622	59.51

**TABLE A4:** Genetic structure characteristics of the black bream compared to other fish species.

Species	Total number of gene	Average transcript length (bp)	Average CDS length (bp)	Average exons number per gene	Average exon length (bp)	Average intron length (bp)
*Megalobrama skolkovii*	32,698	18,474.07	1534.60	8.82	209.69	2124.76
*Danio rerio*	32,077	26,260.74	1685.82	9.36	180.2	2941.3
*Ctenopharyngodon idella*	25,028	19,657.57	1775.93	10.14	175.19	1957.05
*Cyprinus carpio*	43,531	15,584.29	1660.62	9.54	173.99	1629.59
*Megalobrama amblycephala*	29,734	18,783.77	1715.82	9.38	182.97	2037.3
*Anabarilius grahami*	34,414	15,105.52	1309.42	7.86	166.68	2012.35

**TABLE A5:** Number of black bream genes annotated using different databases.

Type		Number	Percent (%)
Annotation	Swissprot	17,839	54.56
	KEGG	9941	30.40
	KOG	23,230	71.04
	GO	28,818	88.13
	NR	29,152	89.16
Total	Annotated	30,558	93.46
	Gene	32,698	—

**TABLE A6:** Evaluating the assembly and completeness of the black bream genome through BUSCO analysis.

Term	Genome BUSCO		Gene BUSCO	
	Count	Ratio (%)	Count	Ratio (%)
Complete BUSCOs	3583	98.5	3497	96.0
Complete single‐copy BUSCOs	3526	96.9	3419	93.90
Complete duplicated BUSCOs	57	1.6	78	2.1
Fragmented BUSCOs	19	0.5	29	0.8
Missing BUSCOs	38	1	114	3.2
Total	3640	—	3640	—

**TABLE A7:** Summary of resequencing data for 49 samples.

Population	Sample	Clean reads (Gb)	Coverage (x)	Mapping rate (%)	Heterozygosity
MA	MA1	23.98	18.95	90.58	0.59
MA	MA2	24.25	19.88	94.03	0.65
MA	MA3	22.26	16.15	83.92	0.60
MA	MA4	23.64	20.66	99.27	0.70
MA	MA5	20.97	18.31	99.40	0.64
MA	MA6	18.7	16.43	99.37	0.40
MA	MA7	22.5	19.72	99.42	0.64
MA	MA8	25.23	22.07	99.28	0.59
MA	MA9	22.09	19.22	99.10	0.69
MA	MA10	26.72	23.44	99.52	0.63
MA	MA11	26.33	23.02	99.43	0.63
MA	MA12	23.78	20.84	99.42	0.72
MA	MA13	31.85	27.91	99.38	0.66
MSpl	MSpl1	22.49	19.95	99.75	0.72
MSpl	MSpl2	23.9	21.18	99.72	0.71
MSpl	MSpl3	23.38	20.80	99.75	0.70
MSpl	MSpl4	29.61	26.18	99.51	0.70
MSpl	MSpl5	23.95	21.29	99.68	0.70
MSpl	MSpl6	23.35	20.75	99.75	0.71
MSpl	MSpl7	30.75	27.12	99.47	0.70
MSpl	MSpl8	22.26	19.76	99.72	0.71
MSpl	MSpl9	23.56	20.90	99.77	0.71
MSpl	MSpl10	39.21	34.62	99.53	0.69
MSpl	MSpl11	38.83	34.26	99.44	0.71
MSpl	MSpl12	33.68	28.12	95.09	0.71
MSpl	MSpl13	37.46	33.07	99.43	0.70
MSpl	MSpl14	39.38	34.60	99.22	0.70
MSpl	MSpl15	38.48	34.16	99.61	0.70
MSnp	MSnp20	23.33	20.72	99.62	0.75
MSnp	MSnp21	23.94	21.22	99.71	0.74
MSnp	MSnp22	22.55	19.92	99.34	0.75
MSnp	MSnp23	23.9	20.97	99.02	0.75
MSnp	MSnp24	22.46	20.00	99.64	0.75
MSnp	MSnp58	19.75	17.11	99.56	0.74
MSnp	MSnp59	24.1	20.35	99.84	0.72
MSnp	MSnp60	21.16	18.48	99.76	0.75
MSnp	MSnp61	26.65	23.66	99.79	0.74
MSnp	MSnp62	25.62	22.42	99.80	0.75
MSnp	MSnp63	25.67	22.83	99.85	0.74
PP	PP1	21.05	19.26	98.79	0.82
PP	PP2	21.05	17.81	99.29	0.82
PP	PP3	20.19	17.17	98.92	0.83
PP	PP4	23.4	20.00	99.29	0.78
PP	PP5	22.69	19.43	99.34	0.81
PP	PP6	23.12	19.85	99.20	0.78
PP	PP7	23.34	20.03	99.21	0.78
PP	PP8	23.52	20.13	99.13	0.72
PP	PP9	22.17	18.67	99.03	0.81
PP	PP10	22.80	17.66	99.20	0.82

**TABLE A8:** Population demographic parameters of black bream and *M. amblycephala*.

Populations	Nucleotide diversity (π)	Tajima's *D*
MSpl	1.19 × 10^−3^	0.834
MSnp	0.97 × 10^−3^	0.472
MA	1.39 × 10^−3^	−0.268

**TABLE A9:** Results of ABBA‐BABA tests.

P1	P2	P3	Dstatistic	*Z*‐score	*p*	f4‐ratio	BBAA	ABBA	BABA
MSpl	MSnp	MA	0.0408	21.6345	2.30E‐16	0.043302	185,804	76712.8	70699.6

## References

[ece370874-bib-0001] Alexander, D. H. , and K. Lange . 2011. “Enhancements to the ADMIXTURE Algorithm for Individual Ancestry Estimation.” BMC Bioinformatics 12: 246. 21682921 10.1186/1471-2105-12-246 PMC3146885

[ece370874-bib-0002] Annaert, P. , B. Swift , J. K. Lee , and K. Brouwer . 2007. “Drug Transporters: Molecular Characterization and Role in Drug Disposition.” Astronomy & Astrophysics 386: 1039–1043.

[ece370874-bib-0003] Banarescu, P. , H. W. Wu , and P. Banarescu . 1967. “The Cyprinid Fishes of China, Vol. I.” Copeia 1967: 491.

[ece370874-bib-0004] Bao, W. , K. K. Kojima , and O. Kohany . 2015. “Repbase Update, a Database of Repetitive Elements in Eukaryotic Genomes.” Mobile DNA 6: 11. 26045719 10.1186/s13100-015-0041-9 PMC4455052

[ece370874-bib-0005] Barton, N. H. , and B. Charlesworth . 1984. “Genetic Revolutions, Founder Effects, and Speciation.”

[ece370874-bib-0006] Benson, G. 1999. “Tandem Repeats Finder: A Program to Analyze DNA Sequences.” Nucleic Acids Research 27: 573–580. 9862982 10.1093/nar/27.2.573 PMC148217

[ece370874-bib-0007] Blanco, E. , G. Parra , and R. GUIGó . 2007. “Using Geneid to Identify Genes.” Current Protocols in Bioinformatics 18: 431–438. 10.1002/0471250953.bi0403s18 18428791

[ece370874-bib-0008] Brower, A. V. Z. 2013. “Introgression of Wing Pattern Alleles and Speciation via Homoploid Hybridization in Heliconius Butterflies: A Review of Evidence From the Genome.” Proceedings of the Royal Society B: Biological Sciences 280: 2302. 10.1098/rspb.2012.2302 PMC3574301 23235702

[ece370874-bib-0009] Carson, H. L. , and A. R. Templeton . 2014. “Genetic Revolutions in Relation to Speciation Phenomena: The Founding of New Populations.” Annual Review of Ecology and Systematics 15: 97–132.

[ece370874-bib-0010] Castresana, J. 2000. “Selection of Conserved Blocks From Multiple Alignments for Their Use in Phylogenetic Analysis.” Molecular Biology and Evolution 17: 540–552. 10742046 10.1093/oxfordjournals.molbev.a026334

[ece370874-bib-0011] Chang, C. C. , C. C. Chow , L. C. Tellier , S. Vattikuti , S. M. Purcell , and J. J. Lee . 2015. “Second‐Generation PLINK: Rising to the Challenge of Larger and Richer Datasets.” GigaScience 4: s13742. 10.1186/s13742-015-0047-8 PMC4342193 25722852

[ece370874-bib-0012] Chen, J. , H. Liu , R. Gooneratne , Y. Wang , and W. Wang . 2022. “Population Genomics of Megalobrama Provides Insights Into Evolutionary History and Dietary Adaptation.” Biology 11: 186. 35205053 10.3390/biology11020186 PMC8869164

[ece370874-bib-0013] Chen, S. , Y. Zhou , Y. Chen , and J. Gu . 2018. “Fastp: An Ultra‐Fast All‐in‐One FASTQ Preprocessor.” Cold Spring Harbor Laboratory 34: i884–i890. 10.1093/bioinformatics/bty560 PMC6129281 30423086

[ece370874-bib-0014] Chen, Y. 1998a. “*Aphyocypris chinensis*: Fauna Sinica Osteichthyes Cypriniformes.”

[ece370874-bib-0015] Chen, Y. 1998b. Fauna Sinica, Osteichthys: Cypriniformes (Part II). Beijing, China: Science Press.

[ece370874-bib-0016] Chen, Y. , L. Zhao , H. Teng , et al. 2021. “Population Genomics Reveal Rapid Genetic Differentiation in a Recently Invasive Population of *Rattus norvegicus* .” Frontiers in Zoology 18: 6. 33499890 10.1186/s12983-021-00387-z PMC7836188

[ece370874-bib-0017] Cheng, H. , E. D. Jarvis , O. Fedrigo , et al. 2022. “Haplotype‐Resolved Assembly of Diploid Genomes Without Parental Data.” Nature Biotechnology 40: 1332–1335. 10.1038/s41587-022-01261-x PMC9464699 35332338

[ece370874-bib-0018] Cheng, P. , D. Yu , Q. Tang , J. Yang , Y. Chen , and H. Liu . 2022. “Macro‐Evolutionary Patterns of East Asian Opsariichthyin‐Xenocyprinin‐Cultrin Fishes Related to the Formation of River and River‐Lake Environments Under Monsoon Climate.” Water Biology and Security 1: 100036.

[ece370874-bib-0019] Clift, P. D. , K. V. Hodges , H. Heslop , R. Hannigan , and G. Calves . 2008. “Correlation of Himalayan Exhumation Rates and Asia Monsoon Intensity.” Nature Geoscience 1: 875–880.

[ece370874-bib-0020] Danecek, P. , A. Auton , G. Abecasis , C. A. Albers , and E. Banks . 2011. “The Variant Call Format and VCFtools.” Bioinformatics 27: 2156–2158. 21653522 10.1093/bioinformatics/btr330 PMC3137218

[ece370874-bib-0021] De Bie, T. , N. Cristianini , J. P. Demuth , and M. W. Hahn . 2006. “CAFE: A Computational Tool for the Study of Gene Family Evolution.” Bioinformatics 22: 1269–1271. 16543274 10.1093/bioinformatics/btl097

[ece370874-bib-0022] Dudchenko, O. , S. S. Batra , A. D. Omer , et al. 2017. “De Novo Assembly of the Aedes Aegypti Genome Using Hi‐C Yields Chromosome‐Length Scaffolds.” Science 356: 92. 28336562 10.1126/science.aal3327 PMC5635820

[ece370874-bib-0023] Durand, N. C. , M. S. Shamim , I. Machol , et al. 2016. “Juicer Provides a One‐Click System for Analyzing Loop‐Resolution Hi‐C Experiments.” Cell Systems 3: 95–98. 27467249 10.1016/j.cels.2016.07.002 PMC5846465

[ece370874-bib-0024] Edelman, N. B. , P. B. Frandsen , M. Miyagi , et al. 2019. “Genomic Architecture and Introgression Shape a Butterfly Radiation.” Science 366: 594–599. 31672890 10.1126/science.aaw2090 PMC7197882

[ece370874-bib-0025] Edelman, N. B. , and J. Mallet . 2021. “Prevalence and Adaptive Impact of Introgression.” Annual Review of Genetics 55: 265–283. 10.1146/annurev-genet-021821-020805 34579539

[ece370874-bib-0026] Ehlers, J. , P. L. Gibbard , and P. D. Hughes . 2018. Quaternary Glaciations and Chronology. Past Glacial Environments. Amsterdam, Netherlands: Elsevier.

[ece370874-bib-0027] Emms, D. M. , and S. Kelly . 2019. “OrthoFinder: Phylogenetic Orthology Inference for Comparative Genomics.” Genome Biology 20: 238. 31727128 10.1186/s13059-019-1832-y PMC6857279

[ece370874-bib-0028] Feng, C. , K. Wang , W. Xu , et al. 2022. “Monsoon Boosted Radiation of the Endemic East Asian Carps.” Science China Life Sciences 66: 563–578. 36166180 10.1007/s11427-022-2141-1

[ece370874-bib-0029] Flanagan, B. A. , S. A. Krueger‐Hadfield , C. J. Murren , C. C. Nice , A. E. Strand , and E. E. Sotka . 2021. “Founder Effects Shape Linkage Disequilibrium and Genomic Diversity of a Partially Clonal Invader.” Molecular Ecology 30: 1962–1978. 33604965 10.1111/mec.15854

[ece370874-bib-0030] Foote, A. D. , N. Vijay , M. C. Avila‐Arcos , et al. 2016. “Genome‐Culture Coevolution Promotes Rapid Divergence of Killer Whale Ecotypes.” Nature Communications 7: 11693. 10.1038/ncomms11693 PMC4895049 27243207

[ece370874-bib-0031] Haas, B. J. , A. L. Delcher , S. M. Mount , et al. 2003. “Improving the Arabidopsis Genome Annotation Using Maximal Transcript Alignment Assemblies.” Nucleic Acids Research 31: 5654–5666. 14500829 10.1093/nar/gkg770 PMC206470

[ece370874-bib-0032] Haas, B. J. , S. L. Salzberg , and W. Zhu . 2008. “Automated Eukaryotic Gene Structure Annotation Using EVidenceModeler and the Program to Assemble Spliced Alignments.” Genome Biology 9: R7. 18190707 10.1186/gb-2008-9-1-r7 PMC2395244

[ece370874-bib-0033] He, S. , H. Liu , Y. Chen , M. Kuwahara , T. Nakajima , and Y. Zhong . 2004. “Molecular Phylogenetic Relationships of Eastern Asian Cyprinidae (Pisces: Cypriniformes) Inferred From Cytochrome b Sequences.” Science in China Series C: Life Sciences 47: 130–138. 15379245 10.1360/03yc0034

[ece370874-bib-0034] Hobolth, A. , J. Y. Dutheil , J. Hawks , M. H. Schierup , and T. Mailund . 2011. “Incomplete Lineage Sorting Patterns Among Human, Chimpanzee, and Orangutan Suggest Recent Orangutan Speciation and Widespread Selection.” Genome Research 21: 349–356. 21270173 10.1101/gr.114751.110 PMC3044849

[ece370874-bib-0035] Hu, J. , J. Fan , Z. Sun , and S. Liu . 2020. “NextPolish: A Fast and Efficient Genome Polishing Tool for Long‐Read Assembly.” Bioinformatics 36: 2253–2255. 31778144 10.1093/bioinformatics/btz891

[ece370874-bib-0036] Hu, X. , P. Luan , C. Cao , et al. 2019. “Characterization of the Mitochondrial Genome of *Megalobrama terminalis* in the Heilong River and a Clearer Phylogeny of the Genus Megalobrama.” Scientific Reports 9: 8509. 31186443 10.1038/s41598-019-44721-2 PMC6559948

[ece370874-bib-0037] Hu, X. , B. Ma , C. Li , et al. 2020. “Genetic Differentiation of an Endangered *Megalobrama terminalis* Population in the Heilong River Within the Genus Megalobrama.” Diversity 12: 404.

[ece370874-bib-0038] Huang, D. , Y. Lin , C. Wan , and Y. Liu . 1997. “Reproductive Biology of the Fish, Megalobrama Skolkovii, in in Fuqiaohe Reservoir.” Acta Hydrobiologica Sinica 21: 15–23.

[ece370874-bib-0039] Hunter, M. E. , N. E. Auil‐Gomez , K. P. Tucker , R. K. Bonde , J. Powell , and P. M. Mcguire . 2010. “Low Genetic Variation and Evidence of Limited Dispersal in the Regionally Important Belize Manatee.” Animal Conservation 13: 592–602.

[ece370874-bib-0040] Jangjoo, M. , S. F. Matter , J. Roland , and N. Keyghobadi . 2016. “Connectivity Rescues Genetic Diversity After a Demographic Bottleneck in a Butterfly Population Network.” Proceedings of the National Academy of Sciences of the United States of America 113: 10914–10919. 27621433 10.1073/pnas.1600865113 PMC5047165

[ece370874-bib-0041] Jiang, H. , Y. Qian , Z. Zhang , et al. 2023. “Chromosome‐Level Genome Assembly and Whole‐Genome Resequencing of Topmouth Culter ( *Culter alburnus* ) Provide Insights Into the Intraspecific Variation of Its Semi‐Buoyant and Adhesive Eggs.” Molecular Ecology Resources 23: 1841–1852. 37475144 10.1111/1755-0998.13845

[ece370874-bib-0042] Jiang, Y. H. , K. H. Han , S. H. Wang , Y. Chen , Y. L. Wang , and Z. P. Zhang . 2018. “Identification and Expression of Transcription Factor sox2 in Large Yellow Croaker *Larimichthys crocea* .” Theriogenology 120: 123–137. 30118947 10.1016/j.theriogenology.2018.07.025

[ece370874-bib-0043] Karin, L. , H. Peter , R. E. Andreas , S. Hans‐Henrik , R. Torbjørn , and D. W. Ussery . 2007. “RNAmmer: Consistent and Rapid Annotation of Ribosomal RNA Genes.” Nucleic Acids Research 35: 3100. 17452365 10.1093/nar/gkm160 PMC1888812

[ece370874-bib-0044] Katoh, K. , and D. M. Standley . 2013. “MAFFT Multiple Sequence Alignment Software Version 7: Improvements in Performance and Usability.” Molecular Biology and Evolution 30: 772–780. 23329690 10.1093/molbev/mst010 PMC3603318

[ece370874-bib-0045] Kim, D. , J. M. Paggi , C. Park , C. Bennett , and S. L. Salzberg . 2019. “Graph‐Based Genome Alignment and Genotyping With HISAT2 and HISAT‐Genotype.” Nature Publishing Group 37: 907–915. 10.1038/s41587-019-0201-4 PMC7605509 31375807

[ece370874-bib-0046] Kong, X. , X. Wang , M. Li , et al. 2021. “Establishment of Myoblast Cell Line and Identification of Key Genes Regulating Myoblast Differentiation in a Marine Teleost, Sebastes Schlegelii.” Gene 802: 145869. 34352298 10.1016/j.gene.2021.145869

[ece370874-bib-0047] Kovaka, S. , A. V. Zimin , G. M. Pertea , R. Razaghi , and M. Pertea . 2019. “Transcriptome Assembly From Long‐Read RNA‐Seq Alignments With StringTie2.” Genome Biology 20: 278. 31842956 10.1186/s13059-019-1910-1 PMC6912988

[ece370874-bib-0048] Kumar, M. , G. Conroy , S. Ogbourne , K. Cairns , L. Borburgh , and S. Subramanian . 2023. “Genomic Signatures of Bottleneck and Founder Effects in Dingoes.” Ecology and Evolution 13: e10525. 37732287 10.1002/ece3.10525 PMC10508967

[ece370874-bib-0049] Kumar, S. , G. Stecher , M. Suleski , and S. B. Hedges . 2017. “TimeTree: A Resource for Timelines, Timetrees, and Divergence Times.” Molecular Biology and Evolution 34: 1812–1819. 28387841 10.1093/molbev/msx116

[ece370874-bib-0050] Lam‐Tung, N. , H. A. Schmidt , V. H. Arndt , and M. B. Quang . 2015. “IQ‐TREE: A Fast and Effective Stochastic Algorithm for Estimating Maximum‐Likelihood Phylogenies.” Molecular Biology and Evolution 32: 268–274. 25371430 10.1093/molbev/msu300 PMC4271533

[ece370874-bib-0051] Li, H. 2023. “Protein‐to‐Genome Alignment With Miniprot.” Bioinformatics 39: btad014. 36648328 10.1093/bioinformatics/btad014 PMC9869432

[ece370874-bib-0052] Li, H. , and R. Durbin . 2009. “Fast and Accurate Short Read Alignment With Burrows‐Wheeler Transform.” Bioinformatics 25: 1754–1760. 19451168 10.1093/bioinformatics/btp324 PMC2705234

[ece370874-bib-0053] Li, H. , B. Handsaker , A. Wysoker , et al. 2009. “The Sequence Alignment/Map Format and SAMtools.” Bioinformatics 25: 2078–2079. 19505943 10.1093/bioinformatics/btp352 PMC2723002

[ece370874-bib-0054] Liu, H. , C. Chen , M. Lv , et al. 2021. “A Chromosome‐Level Assembly of Blunt Snout Bream (*Megalobrama amblycephala*) Genome Reveals an Expansion of Olfactory Receptor Genes in Freshwater Fish.” Molecular Biology and Evolution 38: 4238–4251. 34003267 10.1093/molbev/msab152 PMC8476165

[ece370874-bib-0055] Lomsadze, A. , V. C. Ter Hovhannisyan , O. Yury , and M. Borodovsky . 2005. “Gene Identification in Novel Eukaryotic Genomes by Self‐Training Algorithm.” Nucleic Acids Research 33: 6494–6506. 16314312 10.1093/nar/gki937 PMC1298918

[ece370874-bib-0056] Lowe, T. M. , and S. R. Eddy . 2019. “tRNAscan‐SE: A Program for Improved Detection of Transfer RNA Genes in Genomic Sequence.” Nucleic Acids Research 25: 955–964. 10.1093/nar/25.5.955 PMC146525 9023104

[ece370874-bib-0057] Luo, Y. 1990. “A Revision of Fshes of the Cyprinid Genus Megalobrama.” Acta Hydrobiologica Sinica 14: 160–165.

[ece370874-bib-0058] Ma, X. G. , Y. B. Ren , and H. Sun . 2024. “Introgression and Incomplete Lineage Sorting Blurred Phylogenetic Relationships Across the Genomes of Sclerophyllous Oaks From Southwest China.” Cladistics 40: 357–373. 38197450 10.1111/cla.12570

[ece370874-bib-0059] Majoros, W. , M. Pertea , and S. Salzberg . 2004. “TigrScan and GlimmerHMM: Two Open Source Ab Initio Eukaryotic Gene‐Finders.” Bioinformatics 20: 2878–2879. 15145805 10.1093/bioinformatics/bth315

[ece370874-bib-0060] Malinsky, M. , M. Matschiner , and H. Svardal . 2021. “Dsuite—Fast D‐Statistics and Related Admixture Evidence From VCF Files.” Molecular Ecology Resources 21: 584–595. 33012121 10.1111/1755-0998.13265 PMC7116594

[ece370874-bib-0061] Mao, Y. , C. R. Catacchio , L. W. Hillier , et al. 2021. “A High‐Quality Bonobo Genome Refines the Analysis of Hominid Evolution.” Nature 594: 77–81. 33953399 10.1038/s41586-021-03519-x PMC8172381

[ece370874-bib-0062] Mayr, E. 1954. “Change of Genetic Environment and Evolution as Process.”

[ece370874-bib-0063] Mcginnis, S. , and T. L. Madden . 2004. “BLAST: At the Core of a Powerful and Diverse Set of Sequence Analysis Tools.” Nucleic Acids Research 32: W20–W25. 15215342 10.1093/nar/gkh435 PMC441573

[ece370874-bib-0064] Mckenna, A. , M. Hanna , E. Banks , et al. 2010. “The Genome Analysis Toolkit: A MapReduce Framework for Analyzing Next‐Generation DNA Sequencing Data.” Genome Research 20: 1297–1303. 20644199 10.1101/gr.107524.110 PMC2928508

[ece370874-bib-0065] Meleshko, O. , M. D. Martin , T. S. Korneliussen , et al. 2021. “Extensive Genome‐Wide Phylogenetic Discordance Is due to Incomplete Lineage Sorting and Not Ongoing Introgression in a Rapidly Radiated Bryophyte Genus.” Molecular Biology and Evolution 38: 2750–2766. 33681996 10.1093/molbev/msab063 PMC8233498

[ece370874-bib-0066] Mulder, N. , and R. Apweiler . 2007. “InterPro and InterProScan: Tools for Protein Sequence Classification and Comparison.” Methods in Molecular Biology 396: 59–70. 18025686 10.1007/978-1-59745-515-2_5

[ece370874-bib-0067] Mulian, R. 1994. “Ichthyofauna of the Heilongjiang River.” Chinese Journal of Fisheries 7: 1–14.

[ece370874-bib-0068] Novomodny, G. , P. Sharov , and S. Zolotukhin . 2004. Amur Fish: Wealth and Crisis. Vladivostok, Russia: WWF.

[ece370874-bib-0069] Pastor, T. , J. C. Garza , P. Allen , W. Amos , and A. Aguilar . 2004. “Low Genetic Variability in the Highly Endangered Mediterranean Monk Seal.” Journal of Heredity 95: 291–300. 15247308 10.1093/jhered/esh055

[ece370874-bib-0070] Patra, S. K. , V. Chakrapani , R. P. Panda , C. Mohapatra , P. Jayasankar , and H. K. Barman . 2015. “First Evidence of Molecular Characterization of Rohu Carp Sox2 Gene Being Expressed in Proliferating Spermatogonial Cells.” Theriogenology 84: 268–276. 25913275 10.1016/j.theriogenology.2015.03.017

[ece370874-bib-0071] Pilot, M. , C. Greco , B. M. Vonholdt , et al. 2014. “Genome‐Wide Signatures of Population Bottlenecks and Diversifying Selection in European Wolves.” Heredity 112: 428–442. 24346500 10.1038/hdy.2013.122 PMC3966127

[ece370874-bib-0072] R Core Team . 2011. “R: A Language and Environment for Statistical Computing.” Computing 1: 12–21.

[ece370874-bib-0073] Rivas‐Gonzalez, I. , M. Rousselle , F. Li , et al. 2023. “Pervasive Incomplete Lineage Sorting Illuminates Speciation and Selection in Primates.” Science 380: eabn4409. 37262154 10.1126/science.abn4409

[ece370874-bib-0074] Roques, S. , and J. J. Negro . 2005. “MtDNA Genetic Diversity and Population History of a Dwindling Raptorial Bird, the Red Kite ( *Milvus milvus* ).” Biological Conservation 126: 41–50.

[ece370874-bib-0075] Shen, W. , S. Le , Y. Li , F. Hu , and Q. Zou . 2016. “SeqKit: A Cross‐Platform and Ultrafast Toolkit for FASTA/Q File Manipulation.” PLoS One 11: e0163962. 27706213 10.1371/journal.pone.0163962 PMC5051824

[ece370874-bib-0076] Shultz, A. J. , A. J. Baker , G. E. Hill , P. M. Nolan , and S. V. Edwards . 2016. “SNPs Across Time and Space: Population Genomic Signatures of Founder Events and Epizootics in the House Finch ( *Haemorhous mexicanus* ).” Ecology and Evolution 6: 7475–7489. 28725414 10.1002/ece3.2444 PMC5513257

[ece370874-bib-0077] Simao, F. A. , R. M. Waterhouse , P. Ioannidis , E. V. Kriventseva , and E. M. Zdobnov . 2015. “BUSCO: Assessing Genome Assembly and Annotation Completeness With Single‐Copy Orthologs.” Bioinformatics 31: 3210–3212. 26059717 10.1093/bioinformatics/btv351

[ece370874-bib-0078] Stanke, M. , M. Diekhans , R. Baertsch , and D. Haussler . 2008. “Using Native and Syntenically Mapped cDNA Alignments to Improve De Novo Gene Finding.” Bioinformatics 24: 637–644. 18218656 10.1093/bioinformatics/btn013

[ece370874-bib-0079] Suvorov, A. , B. Y. Kim , J. Wang , et al. 2022. “Widespread Introgression Across a Phylogeny of 155 Drosophila Genomes.” Current Biology 32: 111–123. 34788634 10.1016/j.cub.2021.10.052 PMC8752469

[ece370874-bib-0080] Tang, H. , J. E. Bowers , X. Wang , R. Ming , M. Alam , and A. H. Paterson . 2008. “Synteny and Collinearity in Plant Genomes.” Science 320: 486–488. 18436778 10.1126/science.1153917

[ece370874-bib-0081] Tarailo‐Graovac, M. , and N. S. Chen . 2009. “Using RepeatMasker to Identify Repetitive Elements in Genomic Sequences.” Current Protocols in Bioinformatics 25: 411–414. 10.1002/0471250953.bi0410s25 19274634

[ece370874-bib-0082] Templeton, A. R. 2008. “The Reality and Importance of Founder Speciation in Evolution.” BioEssays 30: 470–479. 18404703 10.1002/bies.20745

[ece370874-bib-0083] Terhorst, J. , J. A. Kamm , and Y. S. Song . 2016. “Robust and Scalable Inference of Population History From Hundreds of Unphased Whole Genomes.” Nature Genetics 49: 303–309. 28024154 10.1038/ng.3748 PMC5470542

[ece370874-bib-0084] Tomasco, I. H. , F. M. Giorello , N. Boullosa , M. Feijoo , C. Lanzone , and E. P. Lessa . 2022. “The Contribution of Incomplete Lineage Sorting and Introgression to the Evolutionary History of the Fast‐Evolving Genus Ctenomys (Rodentia, Ctenomyidae).” Molecular Phylogenetics and Evolution 176: 107593. 35905819 10.1016/j.ympev.2022.107593

[ece370874-bib-0085] Vijay, N. , C. M. Bossu , J. W. Poelstra , et al. 2016. “Evolution of Heterogeneous Genome Differentiation Across Multiple Contact Zones in a Crow Species Complex.” Nature Communications 7: 13195. 10.1038/ncomms13195 PMC5095515 27796282

[ece370874-bib-0086] Wang, C. , L. Yang , Y. Lu , et al. 2024. “Genomic Features for Adaptation and Evolutionary Dynamics of Four Major Asian Domestic Carps.” Science China Life Sciences 67: 1308–1310. 38240907 10.1007/s11427-023-2479-2

[ece370874-bib-0087] Wang, J. , N. R. Street , D. G. Scofield , and P. K. Ingvarsson . 2016. “Variation in Linked Selection and Recombination Drive Genomic Divergence During Allopatric Speciation of European and American Aspens.” Molecular Biology and Evolution 33: 1754–1767. 26983554 10.1093/molbev/msw051 PMC4915356

[ece370874-bib-0088] Wang, Z. , D. Yang , Z. Zhang , X. Ma , X. Jin , and C. Hu . 1993. “The Preliminary Study on Megalobrama Skolkovii Dybowsky From Heilong River.” Fish Heilongjiang S1: 10–13.

[ece370874-bib-0089] Weber, D. S. , B. S. Stewart , and N. Lehman . 2004. “Genetic Consequences of a Severe Population Bottleneck in the Guadalupe Fur Seal ( *Arctocephalus townsendi* ).” Journal of Heredity 95: 144–153. 15073230 10.1093/jhered/esh018

[ece370874-bib-0090] Wolf, J. B. , and H. Ellegren . 2017. “Making Sense of Genomic Islands of Differentiation in Light of Speciation.” Nature Reviews Genetics 18: 87–100. 10.1038/nrg.2016.133 27840429

[ece370874-bib-0091] Yang, Z. 2016. “PAML 4: Phylogenetic Analysis by Maximum Likelihood.” Molecular Biology and Evolution 24: 1586–1591. 10.1093/molbev/msm088 17483113

[ece370874-bib-0092] Yih, P.‐L. 1955. “Notes on *Megalobrama amblycephala*, sp. Nov., a Distinct Species From *M. terminalis* (Richardson).” Acta Hydrobiologica Sinica: 114–122.

[ece370874-bib-0093] Yu, H. , X. Du , X. Li , et al. 2018. “Genome‐Wide Identification and Transcriptome‐Based Expression Analysis of Sox Gene Family in the Japanese Flounder Paralichthys Olivaceus.” Journal of Oceanology and Limnology 36: 1731–1745.

[ece370874-bib-0094] Zhang, C. , S.‐S. Dong , J.‐Y. Wei‐Ming , and Y. Tie‐Lin . 2019. “PopLDdecay: A Fast and Effective Tool for Linkage Disequilibrium Decay Analysis Based on Variant Call Format Files.” Bioinformatics 35: 1786–1788. 30321304 10.1093/bioinformatics/bty875

[ece370874-bib-0095] Zhang, H. H. , M. R. X. Xu , P. L. Wang , et al. 2020. “High‐Quality Genome Assembly and Transcriptome of *Ancherythroculter nigrocauda* , an Endemic Chinese Cyprinid Species.” Molecular Ecology Resources 20: 882–891. 32216061 10.1111/1755-0998.13158

[ece370874-bib-0096] Zhang, W. , K. K. Dasmahapatra , J. Mallet , G. R. P. Moreira , and M. R. Kronforst . 2016. “Genome‐Wide Introgression Among Distantly Related Heliconius Butterfly Species.” Genome Biology 17: 25. 26921238 10.1186/s13059-016-0889-0 PMC4769579

